# Coordinate-Based Lead Location Does Not Predict Parkinson's Disease Deep Brain Stimulation Outcome

**DOI:** 10.1371/journal.pone.0093524

**Published:** 2014-04-01

**Authors:** Kelsey A. Nestor, Jacob D. Jones, Christopher R. Butson, Takashi Morishita, Charles E. Jacobson, David A. Peace, Dennis Chen, Kelly D. Foote, Michael S. Okun

**Affiliations:** 1 Department of Neurology, University of Florida, Center for Movement Disorders and Neurorestoration, McKnight Brain Institute, Gainesville, Florida, United States of America; 2 Department of Neurosurgery, University of Florida, Center for Movement Disorders and Neurorestoration, McKnight Brain Institute, Gainesville, Florida, United States of America; 3 Department of Clinical and Health Psychology, University of Florida, Gainesville, Florida, United States of America; 4 Department of Neurology, Biotechnology and Bioengineering Center, Medical College of Wisconsin, Milwaukee, Wisconsin, United States of America; Philadelphia VA Medical Center, United States of America

## Abstract

**Background:**

Effective target regions for deep brain stimulation (DBS) in Parkinson's disease (PD) have been well characterized. We sought to study whether the measured Cartesian coordinates of an implanted DBS lead are predictive of motor outcome(s). We tested the hypothesis that the position and trajectory of the DBS lead relative to the mid-commissural point (MCP) are significant predictors of clinical outcomes. We expected that due to neuroanatomical variation among individuals, a simple measure of the position of the DBS lead relative to MCP (commonly used in clinical practice) may not be a reliable predictor of clinical outcomes when utilized alone.

**Methods:**

55 PD subjects implanted with subthalamic nucleus (STN) DBS and 41 subjects implanted with globus pallidus internus (GPi) DBS were included. Lead locations in AC-PC space (*x, y, z* coordinates of the active contact and sagittal and coronal entry angles) measured on high-resolution CT-MRI fused images, and motor outcomes (Unified Parkinson's Disease Rating Scale) were analyzed to confirm or refute a correlation between coordinate-based lead locations and DBS motor outcomes.

**Results:**

Coordinate-based lead locations were not a significant predictor of change in UPDRS III motor scores when comparing pre- versus post-operative values. The only potentially significant individual predictor of change in UPDRS motor scores was the antero-posterior coordinate of the GPi lead (more anterior lead locations resulted in a worse outcome), but this was only a statistical trend (p<.082).

**Conclusion:**

The results of the study showed that a simple measure of the position of the DBS lead relative to the MCP is not significantly correlated with PD motor outcomes, presumably because this method fails to account for individual neuroanatomical variability. However, there is broad agreement that motor outcomes depend strongly on lead location. The results suggest the need for more detailed identification of stimulation location relative to anatomical targets.

## Introduction

Parkinson's disease (PD) is a neurodegenerative disorder that impacts multiple motor and non-motor basal ganglia circuits [1], [2]. PD typically manifests between 55 and 65 years, though sufferers can be of any age and also of any ethnicity [1]. The common motor symptoms include resting tremor, rigidity, micrographia, bradykinesia, speech issues, swallowing problems, and difficulties with balance [1]. PD has been associated with non-motor symptoms including sleep disorders, depression, apathy, anxiety, cognitive impairment, and sexual dysfunction [3].

Deep brain stimulation (DBS) is a surgical therapy that has been shown to improve tremor, motor fluctuations, and levodopa-responsive symptoms in a subgroup of well-characterized and carefully screened PD patients [4]–[7]. The subthalamic nucleus (STN) and the globus pallidus interna (GPi) have both been demonstrated to be effective targets for DBS in PD [8], [9]. There are, however, important target-specific differences between STN and GPi DBS. The STN is commonly the preferred DBS target, particularly for younger patients, though this has not been thoroughly tested. Results from studies have revealed that STN DBS improves many of the cardinal motor symptoms of PD (tremor, rigidity, bradykinesia), requires lower energy input when compared to GPi DBS, and facilitates a greater reduction in levodopa requirements [4], [10]–[20]. GPi DBS has been proposed as a potential better target for patients with severe dyskinesia and for those patients with preoperative cognitive or psychiatric dysfunction [21]. Several randomized studies have demonstrated the safety and efficacy of DBS in PD, however, reported outcomes have been widely variable, especially when examining results from individual patients [4]–[7]. A broad spectrum of adverse effects and also benefits has emerged. Declines in verbal fluency associated with STN DBS have, for example, been linked more to a surgical microlesion, rather than to a stimulation-induced effect [22].

The precise anatomic targets that most effectively modulate an individual symptom or symptom complex have not been clearly defined. It has been hypothesized that better motor outcomes might be achieved by applying DBS to specific sub-regions within the STN or GPi targets or by alternatively delivering a therapeutic amount of electrical current to neighboring fiber bundles, rather than stimulating within the targets themselves [21], [23]. A region around the STN, inclusive of an area dorsal to the gray matter target (at the interface of the STN, zona incerta, and Forel's fields) has been shown to be an effective target for stimulation and has been shown to reduce the motor signs of PD in a large case series [23]–[28]. However, targeting this region may not produce optimal outcomes in all patients, with many deriving maximal benefit from stimulation within the borders of the STN itself. Currently, the DBS lead location is most commonly qualitatively derived from a postoperative CT, MRI, or MRI-CT fusion (postoperative CT fused to preoperative MRI) without the use of atlas matching. This qualitatively derived lead location has been used at the bedside to determine the acceptability of the final location of the DBS lead. This bedside methodology, however, is flawed in that it fails to address the proximity of the DBS lead to the many important surrounding structures (e.g. internal capsule, sensory pathways, ocular pathways), and it does not address changes in clinically relevant measures. As imaging and other modalities evolve technologically, we will be able to more specifically target connections within the basal ganglia circuitry (e.g. diffusion tensor imaging (DTI) and tractography). **Table 1** summarizes the many differing methodologies that have been used to derive postoperative lead locations.

**Table 1:** A Summary of Studies Approaching DBS Lead Location and Outcome.

Author	n	Target	Objective	Findings
Walter U, et. al 2011	34	GPi, STN, Vim	To determine if transcranial B-mode sonography (TCS) is a more reliable method than MRI for locating postoperative DBS leads	TCS was not as sensitive in the antero-posterior direction and may not be as accurate as MRI or CT or MR-CT fusion [40].
Schlaier JR, et. al 2012	22	STN	To determine if the use of MER in addition to anatomical targeting would improve clinical outcomes	Intra-operative testing leads physicians to a more favorable final stimulation site than anatomic targeting alone [41].
Lalys F, et.al 2012	30	STN	To identify optimum sites for STN DBS by studying symptomatic motor improvement along with neuropsychological side effects	Suggested a more complete DBS modeling system including lead location, stimulation parameters, and clinical ratings
				Reported good motor improvement from leads located in the STN postero-superior region [42]
Paek SH, et. al 2008	53	STN	To correlate surgical outcome with STN lead position	Reported improvements in all parkinsonian symptoms in all regions of the STN [43]
Connolly PJ, et. al 2012	50	STN	To identify the active contacts and their postoperative location within the STN	Contacts 1 or 2 located in the dorsolateral STN region were active in 90% of patients who underwent DBS one-year prior.
				Stimulating at contact 1 or 2 can decrease initial programming time in the clinic [44]
Thani NB, et. al 2011	8	STN, GPi	To compare measuring lead locations on post-op CT images co-registered to intraoperative MRI versus using an MRI-directed guide tube technique	Concluded that the use of the guided tube technique is accurate in documenting the DBS lead location
				Co-registration of CT images to preoperative MRIs was also considered acceptable [38]
Shahlaie K, Larson PS, Starr PA 2011	15	STN	To study measuring lead location on intraoperative CT (iCT) fused with pre-op MRI in comparison to postoperative MRIs	Lead tip measurements were statistically indistinguishable. iCT can reduce the need for postoperative MRI and avoid the possible complications involved with it [45].
York MK, et. al 2009	18	STN	To correlate lead tip locations, surgery trajectories, and location of active contact with mental status	Declines in mental status score were found in lead locations in the frontal quadrant of both hemispheres.
				Declines in verbal learning were associated with leads that were superiorly located in the left hemisphere but were closer to the STN [46]
Gorgulho AA, et. al 2009	18	STN	To determine the coordinates of the location most likely associated with facial contraction during macrostimulation	Mean *x, y*, and *z* coordinates associated with facial contraction were found to be in close proximity to the internal capsule [47].
Pilitsis JG, et. al 2008	27	Vim	To assess if suboptimal lead location leads to loss of benefit from stimulation in patients with Vim DBS	No significant difference in lead location of those that experienced failure
				Variations in the final lead locations from targets could lead to eventual loss of stimulation efficacy
				Patients with more laterally placed leads experienced worse outcomes [48]
McClelland S 3rd, et. al 2005	26	STN	To measure variations in final lead location from target locations and how these variations impacted clinical efficacy	DBS electrodes placed within a 6 mm diameter cylinder in the predicted center of the STN were associated with similar clinical efficacy [49].
Hamid NA, *et. al* 2005	27	STN	To define the roles of MRI and intraoperative electrophysiological recording in targeting optimum stimulation sites	Because of anatomical variations, fixed pre-determined coordinate targets cannot be applied to all DBS cases [50].
Starr PA, et. al 2006	23	GPi	To identify electrode locations with optimal benefits in patients with dystonia	Mean lead tip and active electrode coordinates did not differ between the group with the best outcomes and the group with the worst outcomes.
				Electrodes with good outcome were near the intercommissural plane [51].

**Legend:** STN- subthalamic nucleus, GPi- globus pallidus internus, VIM- ventralis intermedius nucleus, MER- microelectrode location.

The aim of the current study was to examine whether the precisely measured Cartesian coordinates (*x, y*, and *z* coordinates of the active contact, AC-PC angle, and centerline angle) of an implanted DBS lead are predictive of motor outcome(s) (UPDRS motor ratings) in PD. We aimed to test a methodology that is widely utilized across community and academic centers. We hypothesize that the position and trajectory of the DBS lead relative to the MCP are significant predictors of clinical outcomes. Our expectation was that, due to substantial variation in neuroanatomy among individuals, a simple measure of the position of the DBS lead relative to MCP may not be a reliable predictor of clinical outcomes when utilized alone.

## Methods

### Participants

The study protocol was approved by the Institutional Review Board (IRB) at the University of Florida (UF). The study utilized data from the UF INFORM (Interdisciplinary Florida Registry and Movement Disorders) database of DBS patients. The patients were de-identified in our analysis. Patients gave written approval on informed consent documentation to have their health information recorded and used for purposes of research, as approved by UF IRB.

There were 106 PD subjects drawn from the University of Florida Center for Movement Disorders and Neurorestoration. PD patients were diagnosed utilizing UK Brain Bank criteria and were implanted with unilateral STN or GPi DBS during the period between 2002 and 2012 [29]. Potential candidates were considered appropriate for DBS therapy if they presented with idiopathic PD with an adequate response to dopaminergic medication and at least one of the following: severe on/off fluctuations, disabling “off” time not addressed by medications, severe dyskinesia, and/or a medication-resistant tremor [30]. Patients were screened by a multi-disciplinary team that includes a neurologist, a neurosurgeon, a neuropsychologist, a psychiatrist, a physical therapist, an occupational therapist, and a speech therapist. The team met and discussed surgical candidacy, risks and benefits of DBS therapy, targets, and approaches. Potential candidates were screened using a levodopa/dopaminergic challenge test to determine the response of individual symptoms to standard dopaminergic therapy [31]. Patients were asked to refrain from taking dopaminergic medications twelve hours prior to all neurological evaluations. Motor symptoms in the off-medication state were evaluated using the Unified Parkinson's Disease Rating Scale motor section (UPDRS III). Patients were “challenged” with a suprathreshold dose of dopaminergic medication and then re-tested in an on-medication state. A change score was calculated using the difference between off-medication and on-medication UPDRS III scores [31]. Though a 30% improvement in UPDRS score on a dopamine challenge test has been generally accepted as a minimum requirement for undergoing DBS surgery, patients in this series who failed to meet this response threshold were possibly considered for surgery if the interdisciplinary risk-benefit analysis discussion was favorable and they had one of the following exceptional indications for DBS: severe and debilitating on/off-medication symptom fluctuations that were inadequately measured with UPDRS testing (e.g. disabling dyskinesia) or medication-refractory tremor [31].

All patients were evaluated at the University of Florida Center for Movement Disorders and Neurorestoration, and all surgeries were performed at the University of Florida Hospital. High-resolution postoperative CT scans were performed approximately thirty days following DBS lead implantation to allow for complete resolution of any post-operative pneumocephalus or brain shift.

### Inclusion/Exclusion Criteria

Subjects who underwent unilateral GPi or STN DBS surgery were considered for this study. Within this cohort, subjects were excluded if they were missing any baseline measures or any postoperative UPDRS measures. Systematic and comprehensive pre- and postoperative data collection was performed for each patient. The UPDRS III motor score was collected at each visit and scored by a movement disorders neurologist who had completed the MDS UPDRS training.

### Neurosurgical Procedures

Details of the surgical procedure have been previously published [22]. Briefly, the lead is stereotactically implanted into the brain, and the pulse generator is implanted approximately one month following lead implantation, typically into the subclavicular region.

MRI imaging was performed one day prior to the DBS operation, and a high-resolution, stereotactic CT scan was obtained after application of a stereotactic head ring on the day of surgery. The MRI and CT scan were fused, and a Cartesian coordinate system based on the anterior and posterior commissures was established. A preliminary, indirect target was selected based on typical brain atlas coordinates of the target structure and a safe trans-frontal lobe trajectory to the target was found (avoiding cortical and periventricular veins, sulci and ventricles) by manipulating entry angles. A deformable, three dimensional digital brain atlas was then rotated, translated, and scaled to find a best fit atlas representation of each individual patient's deep brain anatomy using both high-resolution, contrasted MPRAGE images (T1) and FGATIR images (modified inverted T1), which represent deep brain structures much more clearly. The preliminary indirect target and trajectory were then revised to match the desired direct target on the patient's own imaging and the patient-specific deformed atlas [30]. Physiologic confirmation and fine-tuning of the target region was performed using detailed microelectrode recording (MER). Two or more microelectrode passes were typically made to confirm the target structure and identify its critical boundary with the internal capsule [30]. Patients were awake during the surgery and were typically off all antiparkinsonian medications for twelve hours prior to surgery, as the off-medication state provided optimal conditions for using MER and for identifying the typical physiological changes associated with PD. Once a final DBS lead location was selected, the neurosurgeon implanted the DBS lead and the lead position was evaluated with macrostimulation, where stimulation thresholds (the voltage required at standard frequency and pulse width to produce side effects and benefits) were explored and recorded. Two to four weeks after this initial operation, a second procedure was performed where the pulse generator was implanted in the anterior chest wall and connected to the DBS brain lead with a tunneled extension cable [30]. The delay in device activation was to allow edema from the brain implantation to resolve and to eliminate the bias of surgical changes that may have affected tissue impedance and DBS programming [30].

### Image Analysis

Lead locations were measured using a postoperatively acquired, high-resolution CT scan (obtained approximately thirty days post-DBS lead placement to allow for complete resolution of any post-procedure pneumocephalus or brain shift). Postoperative CT images were fused to preoperatively acquired MRI images [32]. This CT-MRI fusion technique has been previously demonstrated to be a very accurate method for identifying the postoperative lead location, since the CT is not subject to the same degree of lead artifact and image distortion as is seen on postoperative MRI studies [32].

The AC-PC based Cartesian coordinate system was used to represent the position of the carefully measured center of the deepest aspect of the deepest cylindrical contact of the DBS lead relative to the MCP. The entry angles (coaxial with the four contact electrode array and ignoring superficial lead trajectory), relative to the AC-PC line sagittally and the median plane coronally, were measured, allowing the simple vector calculation of the position of the centroid of each of the four contacts, based on the known, fixed geometry of the DBS lead. The location of the lead was identified on the CT and projected onto the fused MRI/deformed atlas hybrid to visualize the precise position of the DBS contacts relative to the patient's deep brain anatomy.

The location of the center of each of the four DBS contacts was calculated based on the location of the center of the deepest cylindrical contact of the lead. Typically, only one contact was activated and produced the desired stimulation effect, and the coordinates of this active contact was used in our analysis. When two monopolar contacts were activated simultaneously in an individual patient, the center of the active contact array was calculated as the midpoint between the two cathodal contacts and was used for analysis. The Euclidean distance of each lead from MCP was also calculated using the *x, y, z* coordinates.

### Clinical Outcomes

Pre- and postoperative scores on the UPDRS III were used for the analysis. Preoperative baseline and four-month postoperative scores were compared. Four-month postoperative visits were chosen because clinical measures were systematically collected in both on-medication and off-medication states at that interval.

The Unified Parkinson's Disease Rating Scale Part III (UPDRS III) is a validated measure of motor function in PD. It has fourteen clinician-rated items that include ratings of tremor, bradykinesia, rigidity, postural stability, gait and balance [33]. Items are rated from zero (normal) to four (severely affected) [33].

### Statistics

Patients were grouped separately based on target: STN or GPi. Separate hierarchical linear regressions were conducted among each group. The change in UPDRS motor off-medication scores from baseline to the four-month follow-up were calculated and were used as the dependent variable for each regression. Independent variables in each regression included coordinate data (*x, y*, and *z* coordinates of the active DBS contact) and the AC-PC and centerline angles. Next, coordinate data, AC-PC angle, and centerline angle data were entered into a single model. Hierarchical regressions were computed to examine the effect of this model on the changes in UPDRS motor scores. Finally, the Euclidean distance was entered into a model with the changes in UPDRS scores.

## Results

### Patient Demographics

There were 62 unilateral STN DBS subjects and 44 unilateral GPi DBS subjects identified in the database query. Of this original group, 88.7% (55) of STN DBS subjects and 93.2% (41) of GPi patients met inclusion criteria for the study. There were 16 left GPi DBS, 25 right GPi DBS, 33 left STN DBS, and 22 right STN DBS subjects included. The 10 excluded subjects consisted of 1 rapidly staged bilateral DBS subject (had a second DBS lead implanted before 4-months), 2 subjects lost to follow-up, and 7 subjects who had either missing rating scales or whose evaluations were performed outside the study window. The general characteristics of subjects included in this cohort are summarized in **Table 2**. There were no significant differences at baseline between STN and GPi subjects.

**Table 2:** Baseline Patient Characteristics.

Characteristic	STN (n = 55)	GPi (n = 41)	p value	Entire sample (n = 96)
Age	64.18±8.85	64.55±8.62	n.s.	64.87±8.86
Disease Duration	11.84±6.12	13.44±7.14	n.s.	12.52±5.63
UPDRS (off-med)	39.69±11.52	43.46±11.88	n.s.	41.3±11.76
UPDRS (on-med)	23.35±10.15	25.80±9.10	n.s.	24.4±9.75
H&Y (on-med)	2.78±0.88	2.38±0.42	n.s.	2.32±0.39
H&Y (off-med)	2.75±0.99	2.88±0.87	n.s.	2.81±.73

**Legend:** mean ± standard deviation, UPDRS- Unified Parkinson's Disease Rating Scale, H&Y- Hoehn and Yahr Parkinson's Disease Stage.

### Clinical Outcomes

This study defined improvement as any positive change in score for the UPDRS. Comparing baseline off-medication UPDRS to 4-month off-medication DBS scores revealed a mean improvement of 24.5% in the STN group, and 16.6% in the GPi group. There was no randomization of target selection for this study, and the multi-disciplinary team in general favored the GPi in more complex cases (e.g. cognitive issues, gait problems, significant co-morbidity).

### Lead Location, ACPC & Centerline Angle

The relationship was analyzed between the 4-month active contact coordinates and the patient outcome. The active contact coordinate data was calculated for the STN (**Table 3**) and GPi (**Table 4**) groups. A linear hierarchical regression was performed to determine if patients were more likely to improve based on lead coordinates. For STN (**Table 5**) and GPi (**Table 6**) patients, the *x, y, z* coordinates were not significantly predictive of the patients' UPDRS Off medication- on stimulation score. Additionally, separate hierarchical regression found the AC-PC angle and centerline angle did not contribute to a change in UPDRS score for either the GPi or STN group (all p-values>.1).

**Table 3:** Summary of Active Contact Data in STN Patients.

	*N*	*Minimum*	*Maximum*	*Mean*	*SD*
*x*-coordinate	55	8.94	14.18	11.82	1.37
*y*-coordinate	55	−10.23	3.98	−1.47	2.20
*z*- coordinate	55	−8.65	6.45	−2.60	2.57
ACPC angle	55	46.0	76.0	61.93	5.95
Centerline angle	55	0	27.0	13.06	6.57

**Legend**: *x*-coordinate of the lateral DBS lead position, *y*-coordinate of the antero-posterior DBS lead position, *z*-coordinate of the axial DBS position (all coordinates measured with reference to the MCP- mid-commissural point).

**Table 4:** Summary of Active Contact Data in GPi Patients.

	*N*	*Minimum*	*Maximum*	*Mean*	*SD*
*x*-coordinate	41	17.35	25.78	21.50	1.71
*y*-coordinate	41	−0.07	9.96	3.36	1.88
*z*-coordinate	41	−5.58	3.31	−1.26	1.98
ACPC angle	41	53.0	88.0	64.22	6.37
Centerline angle	41	−5.0	12.0	2.02	4.29

**Legend:** *x*-coordinate of the lateral DBS lead position, *y*-coordinate of the antero-posterior DBS lead position, *z*-coordinate of the axial DBS position (all coordinates measured with reference to the MCP- mid-commissural point).

**Table 5:** Coordinate Analysis of STN Patient.

Change in UPDRS III Motor	F	R^2^	Beta	Sig
** Coordinate Block**	.498	.028		.685
x coordinate			−.107	.447
y coordinate			.159	.386
z coordinate			−.122	.509

**Legend:** UPDRS- Unified Parkinson's Disease Rating Scale, *x*-coordinate of the lateral DBS lead position, *y*-coordinate of the antero-posterior DBS lead position, *z*-coordinate of the axial DBS position (all coordinates measured with reference to the MCP- mid-commissural point).

**Table 6:** Coordinate Analysis of GPi Patients.

Change in UPDRS III Motor	F	R^2^	Beta	Sig
** Coordinate Block**	1.346	.101		.275
x coordinate			−.055	.737
y coordinate			.300	.094
z coordinate			−.244	.162

**Legend:** UPDRS- Unified Parkinson's Disease Rating Scale, *x*-coordinate of the lateral DBS lead position, *y*-coordinate of the antero-posterior DBS lead position, *z*- coordinate of the axial DBS position (all coordinates measured with reference to the MCP- mid-commissural point).

### Model of Predictors

The model of predictors (active contact coordinates, ACPC and centerline angle) was not significantly predictive of change in UDPRS for either the STN (**Table 7**) or the GPI group (**Table 8**). Inspection of individual predictors revealed that, within the GPI group, the y-axis (AP) coordinate (Beta = 0.310, p = 0.082) was the only individual predictor of change in UPDRS motor scores that approached significance. None of the individual predictors approached significance within the STN group.

**Table 7:** Block Analysis of STN Patients.

Change in UPDRS III Motor	FΔ	R^2^Δ	Beta	Sig
**Coordinate & Angle Block**	.309	.031		.905
x coordinate			−.088	.576
y coordinate			.148	.451
z coordinate			−.139	.481
ACPC Angle			−.028	.849
Centerline Angle			−.050	.783

**Legend:** UPDRS- Unified Parkinson's Disease Rating Scale, *x*-coordinate of the lateral DBS lead position, *y*-coordinate of the antero-posterior DBS lead position, *z*-coordinate of the axial DBS position (all coordinates measured with reference to the MCP- mid-commissural point).

**Table 8:** Block Analysis of GPi Patients.

Change in UPDRS III Motor	FΔ	R^2^Δ	Beta	Sig
** Coordinate & Angle Block**	1.357	.168.		.265
x coordinate			−.005	.978
y coordinate			.310	.082
z coordinate			−.294	.114
ACPC Angle			−.269	.114
Centerline Angle			.010	.956

**Legend:** UPDRS- Unified Parkinson's Disease Rating Scale, *x*-coordinate of the lateral DBS lead position, *y*-coordinate of the antero-posterior DBS lead position, *z*- coordinate of the axial DBS position (all coordinates measured with reference to the MCP- mid-commissural point).

### Euclidean Distance

The Euclidean distance from MCP was entered into a linear regression model with change in UPDRS score. There was no significant correlation.

## Discussion

There is broad agreement that the therapeutic effects of DBS are dependent on lead location, but this is not often quantified unless patients experience a negative clinical outcome. However, the benefits of lead localization extend beyond simply troubleshooting poor responders. As illustrated in **Table 1**, many groups have performed lead localization in order to better understand the neuroanatomical targets of DBS. And it is likely that if we could determine location in a quick, easy and uniform way across DBS centers, we could develop a more detailed understanding of the correlation between clinical outcomes and stimulation location. This would be highly desirable for at least two reasons. First, it would facilitate our understanding of why some patients do not have good therapeutic benefit from DBS. Second, it would allow a much more detailed understanding of how clinical outcomes vary as a function of lead location for both motor and non-motor outcomes.

In this paper we examined whether simple measures of lead location (x, y, z coordinates and trajectory angles) were predictors of motor outcomes in PD patients receiving STN DBS. We chose these measures because they can be efficiently computed using imaging acquired as standard care at most DBS centers, and we purposely did not employ complex research methodologies that would be hard to employ in clinical practice. Unfortunately, these simple measures do not provide sufficient predictive power for motor outcomes. We suspect that this is partly because they fail to take into account the considerable neuroanatomical variability that is known to exist among patients, and partly because they fail to account for stimulation location. With regard to the latter, stimulation location is known to vary depending on the details of the stimulation protocol (amplitude, pulse width, frequency, selection of anodes and cathodes). These factors will be further explored in a subsequent study.

Our results indicate that the use of coordinate-based lead location data (*x, y, z* coordinates of the active DBS contact) was not correlated to PD DBS motor outcome. This result was somewhat expected, as the MCP coordinate system does not take neuroanatomical variation between patients into account. It is possible that the combination of coordinate data and voltage data from the active contact may be more predictive of outcome than the use of coordinates alone [34]. Additionally, baseline measures of UPDRS off-medication and the disease duration may also be predictive of outcome, both independent of location and when utilized with lead location data [35]. More sophisticated methods of localizing the center of the active contact may also better correlate location with outcome. Stimulation location, which is distinct from lead location alone, has previously been shown to predict outcome and may be able to provide clinicians with targets more tailored to specific symptoms [23]. It is critical that clinicians remain aware of the many factors, beyond imaging, that may impact outcome.

In the GPi DBS model, the *y*- or antero-posterior coordinate approached but did not reach significance in contributing to clinical outcomes. The relative sizes of the two targets could account for the AP coordinate being important in only GPi DBS. The GPi is much larger than the STN [36]. The results also revealed that as the y-coordinate increased (indicating that the lead was located more anterior in the GPi), the UPDRS III score worsened. This finding is consistent with the literature, which suggests that the posterior-ventral region of the GPi is a preferable site for PD DBS [37]. It is also consistent with the notion that the GPi is a bigger target than the STN (478 mm^3^ versus 158 mm^3^) [36] and therefore may be prone to more error in the final antero-posterior position of the DBS lead. Since the result only trended toward significance, a larger sample size will be needed to confirm this finding.

### Limitations

This study was somewhat limited by the current technology that is available for measuring DBS lead locations. Although demonstrated to be accurate technologies for measuring lead locations, CT-MRI fused images possess potential inaccuracies due to fusion errors or mild effects of artifacts [38].

The study utilized the available data from the UF center, which was unilateral DBS implantations. A future analysis of bilateral cases could possibly yield a better correlation, though it should be noted that DBS outcomes can be more precisely calculated when analyzing only a single lead position.

Lead location measurements were only included from 30 day post-surgery CT scans, and therefore these measurements did not account for potential long-term lead migrations at the time of the 4-month scales. Lead migrations have been reported to occur in 2–3% of cases [39]. Additionally, post-surgical swelling may not be completely resolved 30 days postoperatively, and it is possible, though unlikely, that the final DBS lead location could have been slightly different.

The relative lack of variance among the target coordinate locations may have biased the overall outcomes of this study. The leads in this single experienced DBS center were not surprisingly, placed with little variance. This lack of variance may have impacted the overall power of our dataset to detect changes, especially in the smaller STN target. A follow-up multi-center DBS study may provide us with a larger amount of variance and possibly better results.

## Conclusion

A common approach for examining DBS lead location is based on visual inspection of a postoperative CT, MRI, or CT-MRI fusion. It is much less common to actually measure the post-operative coordinates. These common techniques do not address the proximity of the DBS lead to important surrounding structures (e.g. internal capsule, sensory pathways, ocular pathways), nor do they address changes in clinically relevant outcomes. The current study suggests that utilizing *x, y, z* coordinates, the AC-PC and centerline angles has important limitations. Our group has adopted the use of a three dimensional analysis of the post-operative lead location using a morphed neuroanatomical atlas image. We have observed great inter-individual heterogeneity in neuroanatomy across our cases, and therefore we have used atlas matching, programming data, and UPDRS outcomes to evaluate outcome in our patient population. Graphic representation of lead position relative to neuroanatomical targets through atlas matching, and the use of programming data such as thresholds for stimulation induced side effects, are likely necessary to effectively predict clinical outcomes. The true clinical utility of these factors will be needed to confirm their usefulness in clinical practice.
